# Validity of a Food and Fluid Exercise Questionnaire for Macronutrient Intake during Exercise against Observations

**DOI:** 10.3390/nu11102391

**Published:** 2019-10-07

**Authors:** Floris C. Wardenaar, Daan Hoogervorst, Nancy van der Burg, Joline Versteegen, Wonsuk Yoo, Natasha Tasevska

**Affiliations:** 1 College of Health Solutions, Arizona State University, Phoenix, AZ 85004, USANatasha.tasevska@asu.edu (N.T.); 2 Sports and Exercise Nutrition, Institute for Sports and Exercise, HAN University of Applied Sciences, 6525 Nijmegen, The Netherlands; hoogervorstdaan@gmail.com (D.H.); vanderburgnancy@gmail.com (N.v.d.B.); joline.versteegen@gmail.com (J.V.); 3 Nutrition & Performance, Team Jumbo-Visma, 5222 ‘s-Herthogenbosch, The Netherlands; 4 Global Nutrition Development, Friesland Campina, 3818 Amersfoort, The Netherlands

**Keywords:** nutrition during competition, dietary assessment, self-reporting of intake

## Abstract

Information about the accuracy of self-reported food and fluid intake during competitions is scarce. The objective of this study was to validate a previously developed food and fluid exercise questionnaire (FFEQ) against direct observations made during competitions in athletes. Fifty-eight recreational endurance athletes participating in four different running events and one cross duathlon in the Netherlands between 2015 and 2017 were recruited. The FFEQ overestimated the median energy and carbohydrate intake by 27.6 kcal/h (20.6%) and 9.25 g/h (30.8%) (*p* < 0.001), respectively, compared to direct observation. Reporting bias (i.e., correlation between the difference between methods and average of both methods) increased with a higher energy (*r*: 0.41, *p* < 0.01) and carbohydrate intake (*r*: 0.44, *p* < 0.01). No statistically significant difference was found between FFEQ-reported fluid intake per hour and observations (median difference: −2.93 mL, −1.1%; *p* = 0.48) and no fluid reporting bias was identified (*r*: 0.23, *p* = 0.08). FFEQ-reported energy (*r*: 0.74), carbohydrate (r: 0.74), and fluid (r: 0.85) intake was strongly correlated with the observed intake (all *p*-values < 0.001). In conclusion, the FFEQ accurately estimates the fluid intake on a group level during competitions in recreational athletes. Even though FFEQ overestimates the energy and carbohydrate intake, it is still a useful tool for ranking individuals based on their intake.

## 1. Introduction

There is a broad consensus on the importance of carbohydrate and fluid intake for improving or sustaining exercise performance during endurance exercise [1]. Recommendations for carbohydrate intake range from recommending carbohydrate mouth rinsing during exercise <45 min to the intake of 30–90 g per hour for exercise of a longer duration [2]. For fluid intake, 0.5–2.0 L/h, depending on individual athletes’ needs, is the recommendation [3]. Nowadays, it is generally recommended that fluid losses need to be compensated for or should not exceed 2%–3% of the total body weight, during competitions exceeding 30–45 min [2]. The availability of macronutrient guidelines to improve exercise performance has led to studies investigating whether athletes, individually or in groups, meet these recommendations.

Most studies use self-reporting or observation methods to assess food and fluid and/or macronutrient intake. Regardless of the method used (i.e., observation, questionnaire, food record or recall), a wide range of average intakes for carbohydrate (23.0–84.0 g/h) and fluid (354–765 mL/h) have been reported by athletes during endurance exercise competitions [4,5,6,7,8,9,10,11,12,13,14,15,16,17]. The objective of these studies was to evaluate macronutrient intakes in comparison with the existing nutritional recommendations. It is often thought that athletes may report their dietary intake more accurately than non-athletes, as athletes represent a motivated and disciplined population. Although there is evidence to suggest that motivated subjects report more accurately than less motivated subjects [18], self-reported intake in athletes may still be biased, similar to in other populations [19]. 

We recently developed a questionnaire for measuring food and fluid intake during exercise known as the Food and Fluid Exercise Questionnaire (FFEQ) [10], based on a questionnaire by Pfeiffer et al. [6]. The FFEQ is a 25-item semi-quantitative questionnaire, which includes questions on food and fluid intake, pre-exercise (>1 h before and within 1 h of exercise) and during exercise. This version was recently modified to facilitate the reporting of fluids, and now includes photographs for different serving sizes of beverages [20]. 

There have been a number of dietary validation studies measuring the usual energy intake [21] or daily fluid intake [22] among athletes, but no study so far has investigated the validity of self-reporting methods to measure intake during competitions. Although there are independent biomarkers for energy and sugar intake, doubly labeled water (DLW) measures the energy intake over a multiple-day period, and is not appropriate for assessing the intake over short periods of time [23,24,25]. Urinary sucrose and fructose have been developed as a short-term biomarker of total sugar consumption; however, their performance has not yet been investigated under strenuous exercise protocols [26]. Direct observation, including pre- and post-weighing of consumed food and fluid items, is a useful tool for an objective assessment of intake over short periods of time, especially when participants are being unobtrusively observed in their natural environment [27].

Frequency questionnaires are mainly used to assess food or nutrient intake on a group level, while an individual level of intake may be of interest to professionals providing nutritional advice to athletes for improving their performance during competitions. Self-reported diet is often associated with both random and systematic errors [28], which depend on the dietary assessment method of choice, as well as participants’ characteristics [29]. It is known that biased reporting does not affect the estimated group mean intake, but does affect the precision of the estimates on an individual level. When biased, the distribution of measured intake is artificially widened because of random between-person errors, and systematic and random within-person errors [29], and its usability for evaluating individual intake may thus be limited. An accurate individual assessment of intake during exercise is needed to optimize energy, carbohydrate, and fluid intake during competitions.

To the best of our knowledge, this is the first validation study to estimate the validity of a food and fluid questionnaire that measures the nutrient intake during competitions in athletes. The aim of the present study was to investigate the validity of energy, carbohydrate, and fluid intake assessed by a recently developed web-based FFEQ [20] against well-controlled direct observations made during competitions in recreational endurance athletes.

## 2. Materials and Methods 

### 2.1. Study Design

The validation study collected data from five different endurance events in the Netherlands, i.e., three marathons (42.195 km), a 120 km ultramarathon, and a cross duathlon event (10.5 km running and 20 km of cycling), which took place between December 2015 and March 2017, using direct observations and web-based FFEQ (The Qualtrics Research Suite, 2013. Provo, UT, USA). The study was approved by the Ethical Advisory Board of the HAN University of Applied Sciences (EACO 63.03/17) and performed in accordance with the Declaration of Helsinki. Within seven days before the race, participants completed a baseline questionnaire enquiring about demographics and personal characteristics. On the day of the competition, participants visited our field facility <90 min before the race and within 30 min after the race for instruction and measurements needed for data collection. After the race, all participants received the FFEQ via email, which they had to complete before midnight on the day of the race. 

### 2.2. Participants and Recruitment

A total of 817 athletes (76% men and 24% women) participated in the events that were selected for the purpose of our data collection (three marathons (*n* = 633), 120 km (*n* = 34), and cross duathlon (*n* = 150)). All participating athletes were invited to participate in the study through online study advertisements and newsletters for each of the races. One hundred and six athletes expressed interest in filling out the screening questionnaire. This questionnaire requested information on personal characteristics (sex, age, body height, and weight) and running history, such as individual running goals, weekly hours of running, and running distance (km) per week. No specific exclusion criteria were formulated as long as participants were conditionally capable of participating in the event. All interested cross duathletes were recruited (*n* = 30). Due to the limited research staff capacity during the running events, only interested runners with the fastest personal records for the marathon and ultramarathon were invited to participate (23 out of 63 for the marathon and 8 out of 13 for the 120 km race). In total, 61 participants, aged 18–65 years, participated in the study. 

### 2.3. Procedures

#### 2.3.1. Measurements before and after the Race

All food products and fluids that were brought by the athletes to consume during the race were labeled with a unique participant-specific code and weighed, including wrapping, on a digital scale (Cresta, CKS750, Amsterdam, The Netherlands), before and after the race, with a 0.1 g accuracy. The difference between measurements (pre- and post-race) was calculated to obtain the actual consumed amount of each product (in grams or milliliters). Body weight (kg) was measured before and after the race using a Seca 803 digital scale, and body height was measured before the race on a Seca 213 portable stadiometer.

#### 2.3.2. Measurements during Races

All race organizers allowed runners and cross duathletes to be observed. The selected events allowed runners to be continuously provided with their own food and fluid items during the race, aside from the regular aid stations. Some of the runners received these products from a personal companion that stayed with them during the race, following the runner on a bicycle. If runners had no personal companion, they received their products from our observant that followed the runner during the race on a bicycle. All runners (in the marathon and ultra-marathon) were observed by a research team member on a bicycle, on which an action camera was attached (SJCAM, SJ4000, Shenzhen, China). The observer filmed all consumption while recalling all label product codes on camera and reaffirming the product type and amount (of every product individually). In addition, runners confirmed, on camera, the use of all consumed products and beverages or corrected what was said by the observer. At the same time during recording using the action camera, the observers recorded all foods and fluids consumed by the runner using pre-defined paper forms for which they had been trained. During the race, runners were instructed to hand over all empty bottles, cups, and/or food wrappings to their personal observer after consumption, to be measured after the race. The small number of products provided at aid stations were inventoried before the race, and in case runners consumed such products, the size and type of product was estimated based on the previous inventory, as it was logistically impossible to label these products with a unique code before the race.

Due to the nature of the race course of the cross duathlon, athletes could not be continuously followed during the race, but all their products were labeled and measured pre- and post-race, as described previously. Athletes were instructed to use drop off mats where research team members were available throughout the race to discard their wrappings and leftovers. Seven drop off mats were placed along the route (at 3.5, 7, 13, 15, 23, 25, and 30.5 km), resulting in nine options to drop their consumed products and/or bottles/wrappings, as two drop-off mats were accessible at two points throughout the race. No camera recordings were made during the cross duathlon. 

### 2.4. Food and Fluid Exercise Questionnaire (FFEQ)

**Figure 1:**
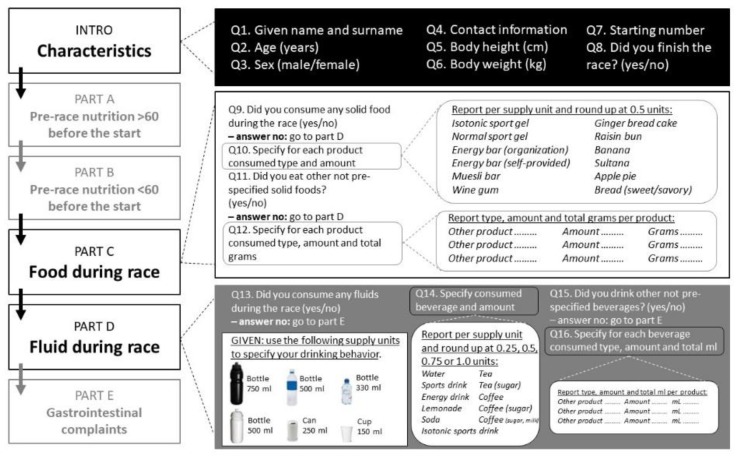
Overview of the food and fluid exercise questionnaire (FFEQ). For the purpose of this study, only responses to the INTRO, part C, and part D were used and validated against observations.

The FFEQ, adapted from Pfeiffer et al. (2009) [6], is a 25-item semi-quantitative questionnaire, including questions on food and fluid intake pre-exercise (>1 h before starting and within 1 h of starting) and during exercise. Compared to the earlier developed questionnaire [10], this version contained photographs of different sizes of bottles, as shown in Figure 1 (see Q13), to help athletes identify and report the size of beverages. The questionnaire takes 10–15 min to complete. It includes six parts: an introduction (INTRO); four parts questioning athletes about their food and fluid intake (Part A: Pre-race nutrition >1 h before the start, including breakfast; Part B: Pre-race nutrition <1 h before the start; Part C: food intake during the competition; Part D: fluid intake during the competition); and one part on gastrointestinal complaints (Part E) (Figure 1). For the purpose of this study, responses to the INTRO, Part C, and Part D were used. Part C started with the following question: “Did you consume any solid food during the race?” (yes/no). If the response was yes, participants were asked to report all consumed products based on a pre-specified list. The pre-specified food list contained 12 items (isotonic sports gel, energy gel, energy bar provided by the race organization, self-provided energy bar, muesli bar, gingerbread cake (slice), raisin bun, banana, sultana (in the Netherlands, a popular low-fat, high-carb cookie wrapped per three pieces), apple pie, “chewables” such as wine gums (per piece), bread with sweet spread, and bread with savory cuts or spread). Participants were then asked if they had consumed any other not pre-specified solid foods during the race. If they responded “yes”, participants were asked to list up to three different options of other products, identifying the type (and brand, if available), total number, and total grams, if known. Part D started with the following question: “Did you consume any fluids during the race?” (yes/no). If the response was yes, participants were asked to report all consumed fluids based on a pre-specified list containing 11 items. They were asked to report their fluid intake based on a choice of common serving types and sizes and to specify the total amount consumed (e.g., 750 mL bottle (water, isotonic sports drink, energy drink, lemonade), 500 mL bottle (water, isotonic sports drink, energy drink, lemonade), 330 mL (water, isotonic sports drink, energy drink, lemonade), soda can (250 mL, soda, energy drink), cup (150 mL, water, tea, tea with sugar, coffee, coffee with sugar, coffee with milk, coffee with milk and sugar)) (Figure 1). Finally, participants were asked if they had consumed any other fluids during the race. If the answer was yes, participants were able to list up to three different options of other products, specifying the type (and brand, if available), total number, and total milliliters (mL), if known. The FFEQ is available in Dutch, though an English translation can be provided upon request.

### 2.5. Calculation of Food and Fluid Intake, and Estimation of Energy and Macronutrient Intake

For both the observation and the FFEQ, the total energy (kcal), carbohydrate (CHO, g), and fluid (mL) intake was calculated using product label declarations or the Dutch food composition database (Dutch Food Composition Database version 2016/5.0, NEVO). Calculations for the *observation* were based on the total product consumption (grams or milliliters) values that were obtained using pre- and post-race measurements on a precision scale. The FFEQ calculations were based on the total registered product size (number of servings and/or weight or volume) that were reported by the participants. All calculations were performed using a pre-defined calculation format with standardized reference products, as shown in Table 1. Reference product nutrient declarations were based on the NEVO table for common foods, such as muesli bars, wine gums, ginger bread cake (ontbijtkoek in Dutch), raisin buns (krentenbol in Dutch), bananas, sultanas, apple pie, and bread with sweat or savory spread [30].

At least three common sport foods were used to generate averages for energy, carbohydrate, and fluid, for obtaining a representative reference product for isotonic sport gels, normal sport gels, and two energy bar options. Participants were able to choose from the pre-specified food options, or they could opt for the option “other” and enter the type of product they used, including the brand name. The calculations were performed by a trained sport dietitian and were independently checked by another research team member.

### 2.6. Data Analysis

Statistical analysis was carried out per-protocol set analysis for the data of subjects who completed the race, had undergone complete observation, and completed the FFEQ by midnight of the day of the race, using statistical software program SPSS (version 25). Personal characteristics are presented as percentages or the mean ± standard deviation (SD). As values for energy and carbohydrate and fluid intake were not normally distributed in all subgroups, medians and interquartile ranges (IQR) are presented. Because of the difference in exercise time between the events, intake was expressed as kcal, g, or mL per hour. We analyzed men and women combined, as the mean difference between the FFEQ-reported and observed intake for energy, CHO, and fluid did not significantly differ between men and women (Mann–Whitney U test, *p* > 0.436 for energy, CHO, and fluid). Differences in intake estimates between the observations and FFEQ were assessed using Wilcoxon signed-rank tests. To investigate the ranking of individuals according to their intake, partial Spearman correlation coefficients adjusted for sex, BMI, and running speed were used, and 95% CI were calculated using Fisher’s Z transformation. Bland–Altman plots were generated to evaluate the agreement between the FFEQ-measured and observed energy, macronutrient, and fluid intake. For this plot, the difference between the two methods was plotted against the mean intake by the two methods. We added 95% limits of agreement (mean ± 2SD) to the plot. Furthermore, Spearman correlations were calculated between the mean of the two methods and the difference between the two methods, to assess the significance of the slope of misreporting. Finally, we conducted stratified exploratory analyses and examined differences in energy, carbohydrate, and fluid intake between the two methods by event, pre-race BMI, speed (distance in km/hour), and type of products consumed (classified as participants consuming carbohydrate-containing liquids, (additional) solid foods, or only water). 

We performed a posteriori sample size calculation based on (1) the 95% limits of agreement for the mean difference between the FFEQ data and observations of the Bland–Altman analysis (Lu et al. 2016; Bland et al., 1986), and (2) identification of a difference based on a paired t-test between two means (Dawson-Saunders et al. 1990). For our study sample of 58, we had an 84% power to detect the agreement based on the maximum allowable difference of 46 g/h using differences in the mean (11.1 g/h) and standard deviation (18.1 g/h) for CHO data with a two-sided 5% significance level. For the t-test, a sample of 23 was necessary to satisfy at least 80% of the power with the two-sided 5% significance level. Both approaches showed that our study sample of 58 has more than an 80% power to identify that the methods are in good agreement. 

All statistical analyses were performed with significance levels set at *p* ≤ 0.05.

## 3. Results

Data were collected from 58 participants (*n* = 47 male and *n* = 11 female), as shown in Table 2. Three of the participants involved in the 120 km competition dropped out: one dropped out due to an injury and two stepped out at a turning point because they were behind on the organizational slowest allowable time schedule. The participants had an average age of 43.0 ± 9.3 years, body height of 180 ± 7.6 cm, and body weight of 73.6 ± 8.7 kg. The subjects competed in three types of events, with the following durations expressed as the median and interquartile range (IQR): marathon (*n* = 23, duration 3:38 (3:21–4:09) hours), ultramarathon (*n* = 5, duration 13:08 (10:38–13:34) hours), and a cross duathlon (*n* = 30, duration 2:17 (2:00–2:27) hours).

### 3.1. Energy, CHO, and Fluid Intake Based on Observation

The median energy intake (IQR) based on direct observation for all participants was 134 (73.1–189) Kcal/h, with a CHO intake of 30.0 (16.7–44.2) g/h and a fluid intake of 262 (173–375) mL/h (Table 3). These totals were based on the combination of two different sports (cross duathlon and running) and different distances. By event, energy, CHO and fluid intake per hour were the lowest during the cross duathlon, and greatest during the 120 km race.

### 3.2. Energy, CHO, and Fluid Intake Based on FFEQ

Based on the FFEQ, the median energy intake (IQR) for the whole group was 153 (90.4–259) Kcal/h, with a CHO intake of 36.9 (21.6–63.6) g/h and a fluid intake of 268 (172–417) mL/h. By event, the cross duathlon group reported the lowest values for energy, CHO, and fluid, followed by the 120 km group, while the marathon group reported the highest values for all exposures of interest, as shown in Table 3.

### 3.3. Comparison of Methods

#### 3.3.1. Group Difference between Methods

On a group level (Table 3), the intakes of energy and carbohydrate (CHO) were significantly over-reported (*p* < 0.001) when using the FFEQ compared to direct observation; the median difference (IQR) was 27.6 (−15.3 to 96.7) kcal/hour or 20.6% of the median for energy, and 9.25 (−0.17 to 23.4) g/hour or 30.8% for CHO. No statistically significant difference was found between the observed and FFEQ-reported fluid intake per hour (*p* > 0.05), with a median difference of −2.93 mL/h (−40.9 to 57.2). Assessing the mean difference between methods by event shows that the participants in the marathon distance event over-reported energy by 38% (*p* < 0.001), CHO by 32% (*p* < 0.001), and fluid by 19% (*p* < 0.05). Although the 120 km runners under-reported energy and CHO and over-reported fluid, neither of the three differences reached significance (*p* > 0.05), while the duathlon runners only slightly over-reported CHO by 2% (*p* < 0.05).

Table 4 shows the results from stratified analyses by BMI, speed, and categorized food and fluid intake. In the analysis of BMI, those with a BMI >22.5 reported higher energy and carbohydrate intakes, with a mean difference of 42.6 (2.13 to 106) kcal/h (*p* < 0.001) and 10.8 (1.35 to 23.7) g/h (*p* < 0.001), respectively. Participants with a lower BMI (<22.5) reported a significantly higher intake of CHO (5.20 (−5.94 to 23.1) g/h (*p* < 0.05)), but not energy. In the analysis of speed, those with a speed <12.4 km/h reported a higher energy and carbohydrate intake with the FFEQ compared to the observed intake, with a difference of 52.0 (−7.62 to 130) kcal/h (*p* < 0.05) and 10.3 (0.31 to 24.9) g/h (*p* < 0.05), respectively, whereas the faster participants only over-reported the CHO intake (7.28 (−1.83 to 18.9) g/h) (*p* < 0.05) and not the energy intake (*p* > 0.05). Finally, categorizing participants into those consuming fluids with energy (such as sports drinks and energy gels and water), fluids and solid foods, or water only, resulted in over reporting of the fluids for the energy group only. This group over-reported both energy (21.7 (−20.1 to 84) kcal/h) and carbohydrate (9.53 (−2.93 to 23.2) g/h) (*p* < 0.001), and slightly under-reported the fluid intake (-5.16 (−40.1 to 50.3) mL/h) (*p* < 0.05). No significant differences were found for the fluids and solids and water only groups in reporting energy, CHO, and fluids (*p* > 0.05). 

#### 3.3.2. Ranking of Individuals According to Intake

The correlation between observed and FFEQ-reported energy was 0.74 (95%CI: 0.58–0.84) for energy and 0.74 (95%CI: 0.56–0.84) for CHO. The correlation for fluids was strongest, with r = 0.85 (95%CI: 0.75–0.91). Correlations between self-reported and observed intake remained statistically significant for all exposures at *p* < 0.001 among marathon and duathlon participants only, with r-values ranging from 0.73 to 0.81 (Table 3). In stratified analyses (Table 4), correlations in all subgroups were significant (*p* ≤ 0.05). Correlations remained strong for energy, CHO, and fluid in those with a BMI >22, with r ranging from 0.77 to 0.87, and a speed >12.4 km/h, with r ranging from 0.77 to 0.88. 

#### 3.3.3. Agreement between Methods

**Figure 2:**
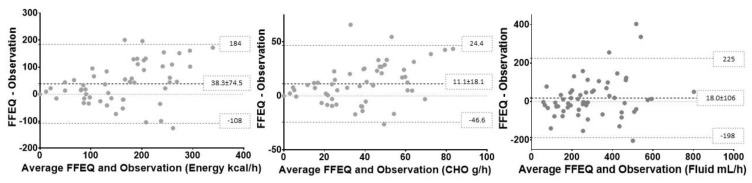
Bland–Altman figures for energy (kcal/h), carbohydrate (CHO) (g/h), and fluid intake (FLU) (mL/h), including 95% limits of agreement.

Bland–Altman plots for energy, carbohydrate, and fluid intake per hour for the whole group are shown in Figure 2. On average, the energy intake was over-reported on the FFEQ by 20.6% or 38.3 ± 74.5 kcal/h, with a 95% limit of agreement between -108 to 184 kcal/h. On average, the carbohydrate intake was over-reported on the FFEQ by 30.8% or 11.1 ± 18.1 g/h, with a 95% limit of agreement from -46.6 to 24.4 g/h. Finally, on average, fluid consumption was over-reported on the FFEQ by −1.1% or 18.0 ± 106 mL/h, with a 95% limit of agreement from -198 up to 225 mL/h.

Correlations between the difference and the means of the observed and FFEQ-reported intake were 0.41, *p* > 0.01, for energy; 0.44, *p* > 0.01, for carbohydrate; and 0.23, *p* = 0.08, for fluid intake. This shows that for both energy and carbohydrate, there was a significant bias in reporting. These moderate positive correlations showed a higher level of energy and carbohydrate over reporting on the FFEQ with higher amounts consumed. We found no evidence of reporting bias in FFEQ-reported fluid intake (*p* > 0.05). 

## 4. Discussion

To the best of our knowledge, this is the first validation study comparing a dietary assessment instrument to assess intake against observations during sport competitions. On average, the FFEQ over-reported energy and CHO intake by 25%–30%, and the level of misreporting was greater at higher intakes. For fluids, the FFEQ-reported intake was similar to the observed intake on a group level, without reporting bias. We found a strong correlation between the FFEQ-reported and observed intake for all three exposures of interest, suggesting that the FFEQ is still a useful tool for ranking individuals based on their intake. Our exploratory analysis on potential determinants of misreporting showed that BMI, running speed, and type of products may influence the level of misreporting. 

Twenty-eight participants included in this study were part of a larger dataset comparing GI complaints and the dietary intake of runners competing at different distances [20]. The dietary intake of these 28 runners that completed the FFEQ and were observed was on the lower end of the reported intake of the larger group of runners that only completed the FFEQ (*n* = 158), who reported a median energy intake of 200 (139–291) kcal/h, a carbohydrate intake of 42.1 (31.1–63.3) g/h, and a fluid intake of 358 (245–78) mL/h [20]. This suggests that the level of misreporting may have been underestimated in our group of 28 participants, as they were aware that they were being continuously observed. When comparing events, marathon runners substantially over-reported their CHO intake compared to cross duathletes, who were observed in a different way. Another reason for the difference in reporting may be that while both the marathon and cross duathlon participants were able to bring their own products, the marathon events had more aid stations (every 5 km). The use of products from aid stations may have resulted in difficulties in estimating the serving size, resulting in misreporting. Although the 120 km group under-reported, due to the small numbers, the difference between the FFEQ-measured and observed intake did not reach significance. 

Despite the relatively low reported intakes, the athletes in this study still over-reported energy and carbohydrate on the FFEQ. In contrast to our findings, a meta-analysis of 11 studies comparing the self-reported energy intake to energy expenditure assessed via DLW showed an under reporting of energy intake [21]. The mean energy intake was under-reported by 19% (−2793 ± 1134 kJ/day) [21]. Most of the included studies used a 4–7 day food record and the percent of under reporting ranged from 1% to 34% [21]. The FFEQ was specifically designed for athletes, while in other studies, conventional dietary assessment methods were applied to athletes. These methods do not take into account the characteristics of an athlete’s diet [21], such as the inclusion of sport nutrition products [31], or give the option to report pre-defined products that are often used during exercise. The over reporting in the current study may have been due to (1) a large percentage of athletes reporting the use of energy gels, which, most of the time, were not completely consumed, resulting in an overestimation of the intake, and (2) instructing participants to report solid foods at 0.5 unit increments, which may have led to the over reporting of energy and carbohydrate. Despite CHO intake being over-reported on the FFEQ, the observed median intake was at the suggested carbohydrate recommendation of 30 g/h, meaning that 50% of the group met the lower end of the recommended intake for endurance athletes [32]. Surprisingly, the estimation of fluid intake, regardless of using transparent or non-transparent drinking bottles, did not lead to misreporting on a group level. This may be, at least partly, due to instructing participants to round up the unit of measurement to 0.25 units and providing a wide variety of serving sizes with pictures.

Sports dietary assessment is routinely conducted for evaluating what an athlete consumes [33]. Sport dietitians mainly use retrospective methods to obtain more insight into athletes’ dietary behavior during competitions [33]. As part of dietary counselling, recalling the intake over a certain period of time (<24 h) is most commonly used. This approach is sometimes used in combination with pictures taken by the athletes about the food they have consumed. Assessing nutrient intake in a digitalized standardized way is a new type of nutritional service offered to individual athletes [34]. Theoretically, a pre-specified questionnaire, such as the FFEQ, could standardize and simplify the way intake data are collected by athletes. The strong correlations found between the FFEQ and observations make the tool suitable to differentiate within a group or team athletes with low and high intakes. At the same time, we found that the FFEQ provided an accurate estimate of fluid consumption on a group level, but failed to accurately assess energy and carbohydrate intake due to over reporting and reporting bias at higher intakes. As such, and because of the large variation of the reporting accuracy in individuals, this type of method should be used with caution on an individual level. While the mean intake on a group level may reflect the actual mean intake, a substantial portion of the individual estimates may be inaccurate [29]. Future research should focus on test-retest variability for this type of method as this is critical for assessing the habitual intake during exercise over time [21]. It should be taken into account that repeated use of the FFEQ, for example, during multiple stages of events such as the Tour de France, may influence the quality of reporting, as the participants’ ability to recall their intake may improve with time [31]. 

FFEQ-reported intake in the current study is similar to the previously reported macronutrient intake during endurance events. The median carbohydrate intake was 36.9 (21.6–63.6) g/h, which fits within the range of carbohydrate intakes during endurance events (i.e., running, triathlon) reported by others (23.0–84.0 g/h) [4,5,6,7,8,9,10,11,12,13,14,15,16,17]. The fluid intake of 268 (172–417) mL/h was relatively low compared to the 354–765 mL/h reported by others during competitions [4,5,6,7,8,9,10,11,12,13,14,15,16,17], possibly because our data were collected during winter. As many endurance athletes use sport drinks as the main source of carbohydrate consumption, this may have affected the carbohydrate intake. The FFEQ was designed to record the dietary intake during exercise. The results of the current study show that the FFEQ is associated with reporting bias for energy and CHO. Still, it is unknown if the level of bias in other methods used in sporting events, such as food records or face-to-face recall, is similar. Studies using food records reported slightly higher mean intakes for carbohydrate (44–50 g/h) and fluid (415–765 mL/h) than the current study [13,14]. The measurement error due to half-emptied gels and difficulty in estimating leftovers in non-transparent bottles would be similar across all methods, although one can assume that this type of error would be random. The FFEQ method relies on memory, whereas in some studies, runners were asked to recall the intake at multiple time points during the race (resulting in a shorter recall period) or to record food and fluid intake using pen and paper during exercise [14,35]. Nonetheless, methods such as multiple face to face recalls during a race or a food record at aid stations, are less feasible for performance-focused athletes participating in competitive events.

We believe that using unobtrusive observations is the best available validation approach, though errors in observation- and FFEQ-derived energy and CHO estimates may be correlated, as both approaches rely on Food Composition Tables to generate intake. There are independent biomarkers for food and fluid intake (i.e., energy, carbohydrates, and fluid), yet DLW cannot be used to assess the intake over several hours [21]. The performance of urinary sucrose and fructose, a short-term biomarker of total sugar consumption, has not yet been investigated under strenuous exercise protocols [26]. 

Our study has certain limitations. The FFEQ did not take timing of food consumption into account, while based on previous reporting, we know that food intake during the course of the race may differ [5,14]. Especially during longer races, this may be seen as a disadvantage [33,36]. In this study, one trained dietitian interpreted and calculated all data to avoid any source of variability when processing the data [37]. Observational data collection differed between events. While all runners were continuously observed by a cyclist collecting all consumed products, we used drop-off areas during the cross duathlon to collect these items. However, the rest of the data collection and measurements were similar across all events. Observation may alter the diet of the observed [33], but it seems unlikely that athletes change their behavior during competitions. Even though, in our stratified analysis, the subgroups had a rather small sample size, our posteriori sample size calculation showed that we needed at least 23 participants to obtain the required 80% power to detect differences between means using a paired t-test with a two-sided 5% significance level. Only a few of the subgroups had a sample size <23 and needed to be interpreted with caution. Given the limited number of female participants in our study population (*n* = 11), we have not reported a stratified analysis by sex and, thus, we were unable to investigate sex differences in misreporting.

Finally, the validity of results may have been compromised by the non-randomized selection of participants [21]. Inclusion in the study was voluntary and the incentive was modest. Therefore, we were likely to recruit participants that were already interested in the topic and our data may not reflect an accurate assessment of athletes that are not primarily interested in sport nutrition. 

## 5. Conclusions

In conclusion, the FFEQ reports an accurate fluid intake, but over reports the energy and carbohydrate intake, on a group level during competitions in recreational athletes. On an individual level, the FFEQ may over or under report, but it is still a useful tool for ranking individuals based on their intakes of energy, carbohydrate, and fluid. 

## Figures and Tables

**Table 1:** Quantity, energy, carbohydrate, and fluid content of pre-specified foods and beverages listed in the FFEQ.

Product	Standard Quantity	Energy (Kcal)	CHO (g)	Fluid (mL)
**Solid Foods Per Supply Unit:**				
Isotonic sports gel	60 g	92	22.0	30
Energy sports gel	60 g	117	29.0	10
Energy bar (organization)	45 g	193	36.0	0
Energy bar (self-provided)	45 g	193	36.0	0
Muesli bar	25 g	129	16.0	0
Gingerbread (slice)	30 g	172	39.0	0
Raisin bun	50 g	134	25.0	0
Banana	130 g	123	27.0	0
Sultana	40 g	157	30.0	0
Apple pie	115 g	292	41.7	0
Chewables (e.g., wine gums)	5 g	16	3.7	0
Bread with sweet spread	40 g	133	27.3	0
Bread with savory cuts or spread	40 g	124	15.6	0
**Fluids per 100 mL:**				
Water	100 mL	0	0	100
Isotonic sports drink	100 mL	33.2	5.8	100
Energy drink	100 mL	60.8	15.0	100
Lemonade	100 mL	41	10.0	100
Soda	100 mL	25.2	6.4	100
Tea	100 mL	0	0	100
Tea with sugar	100 mL	10.7	2.7	100
Coffee	100 mL	1	0.1	100
Coffee with sugar	100 mL	11.7	2.8	100
Coffee with milk	100 mL	12	1.3	100
Coffee with milk and sugar	100 mL	28	5.3	100

**Table 2:** Characteristics of the study participants.

	Sex		Age (y)	Height (cm)	Body Weight (kg)	BMI	Speed (km/h)	Distance (*n*)		
	*n*	%	Mean ± SD	Mean ± SD	Mean ± SD	Mean ± SD	Mean ± SD	Marathon	120 km	Duathlon
Male	47	81	44.2 ± 8.7	182 ± 6.5	76.0 ± 7.4	23.0 ± 1.9	12.3 ± 2.2	19	5	23
Female	11	19	37.6 ± 10.6	173 ± 8.3	63.4 ± 6.2	21.1 ± 1.5	12.4 ± 1.2	4	0	7
Total	58	100	43.0 ± 9.3	180 ± 7.6	73.6 ± 8.7	22.6 ± 2.0	12.3 ± 2.0	23	5	30

**Table 3:** Median and interquartile range (IQR) of observed and FFEQ-measured energy (kcal), carbohydrate (CHO), and fluid (FLU) intake per hour, difference, and correlations with a 95% CI between FFEQ-measured and observed intake for all participants and per event.

	*n*	Energy (Kcal/h)					CHO (g/h)					Fluid (ml/h)				
		OBS	FFEQ	Diff.	*r*	95%CI	OBS	FFEQ	Diff.	*r*	95%CI	OBS	FFEQ	Diff.	*r*	95%CI
**Total**	58	134 (73.1; 189)	153 (90.4; 259)	27.6 * (−15.3; 96.7)	0.74 *	0.59; 0.84	30.0 (16.7; 44.2)	36.9 (21.6; 63.6)	9.25 * (−0.17; 23.4)	0.74 *	0.58; 0.84	262 (173; 375)	268 (172; 417)	−2.93 (−40.9; 57.2)	0.85 *	0.75; 0.91
Marathon	23	154 (94.9; 198)	248 (139; 314)	95.0 * (33.4; 129)	0.81 *	0.60; 0.92	35.3 (21.9; 48.3)	51.6(33.3; 67.1)	14.8 * (4.80; 27.0)	0.76 *	0.51; 0.89	327 (224; 410)	405 (258; 521)	49.6^α^ (−19.3; 113)	0.73 *	0.46; 0.88
20 km	5	254 (137; 274)	188 (144; 237)	−36.2 (−102; 58.0)	−0.3	−0.93; 0.79	43.7 (25.4; 62.5)	36.2 (27.8; 49.1)	−16.7 (−21.7; 15.2)	0.2	−0.83; 0.92	400 (324; 538)	399 (367; 418)	19.0 (−131; 42.9)	0.30	−0.79; 0.93
Duathlon	30	103 (37.1; 155)	99.3 (50.0; 200)	11.2 (−16.9; 44.1)	0.81 *	0.64; 0.91	25.4 (5.47; 35.4)	26 (14.5; 61.9)	6.3 ^α^ (−1.26; 18.2)	0.76 *	0.55; 0.88	186 (144; 264)	208 (114; 275)	−11.9 (−44.9; 14.5)	0.78 *	0.58; 0.89

Significant values for r indicated with symbols: * ≤0.001 and ^α^ ≤0.05.

**Table 4:** Median and IQR, difference, and correlations with a 95% CI between FFEQ-measured and observed intake for energy, carbohydrate (CHO), and fluid (FLU) per hour by BMI, speed, and intake.

BMI	*n*	Energy (Kcal/h)					CHO (g/h)					Fluid (ml/h)				
		OBS	FFEQ	Diff.	r	95%CI	OBS	FFEQ	Diff.	r	95%CI	OBS	FFEQ	Diff.	r	95%CI
<22.5	29	140 (70.5; 224)	156 (84.5; 252)	9.20 (−35.5; 70.8)	0.51 ^α^	0.18; 0.74	33.7 (9.20; 48.4)	37.7 (17.9; 63.5)	5.20 ^α^ (−5.94; 23.1)	0.59 ^α^	0.29; 0.79	263 (178; 385)	258 (170; 401)	−19.3 (−63.9; 48.5)	0.79 *	0.60; 0.90
>22.5	29	132 (73.3; 170)	151 (91.3; 270)	42.6 * (2.13; 106)	0.87 *	0.74; 0.94	29.9 (18.0; 41.0)	36.1 (21.9; 63.7)	10.8 * (1.35; 23.7)	0.77 *	0.56; 0.89	257 (158; 378)	275 (169; 496)	12.9 (−14.6; 109)	0.80 *	0.61; 0.90
**Speed (km/h)**																
<12.4	29	132 (70.8; 188)	188 (100; 270)	52.0 ^α^ (−7.62; 130)	0.54 ^α^	0.22; 0.76	27.1 (15.6; 44.7)	36.2 (21.8; 64.9)	10.3 ^α^ (0.31; 24.9)	0.50 ^α^	0.16; 0.73	280 (180; 405)	335 (191; 497)	17.8 (−28.6; 78.0)	0.77 *	0.56; 0.89
>12.4	29	137 (73.0; 197)	146 (74.0; 228)	14.3 (−18.0; 50.2)	0.87 *	0.74; 0.94	33.7 (15.0; 46.6)	37.7 (19.0; 63.3)	7.28 ^α^ (−1.83; 18.9)	0.88 *	0.76; 0.94	255 (161; 330)	246 (147; 351)	−8.35 (−58.8; 54.1)	0.77 *	0.56; 0.89
**Intake**																
Fluids with energy	35	141 (89.0; 189)	153 (92.1; 267)	21.7 * (−20.1; 84.0)	0.67 *	0.43; 0.82	32.9 (21.4; 45.8)	41.8 (22.3; 65.6)	9.53 * (−2.96; 23.2)	0.66 *	0.42; 0.81	257 (171; 384)	258 (162; 426)	−5.16 ^a^ (−40.1; 50.3)	0.88 *	0.77; 0.94
Fluids & solids	19	140 (80.6; 205)	194 (102; 257)	54.5 (−115.1; 129)	0.43 ^α^	−0.03; 0.74	33.7 (19.7; 48.3)	45.8 (21.8; 60.7)	12.9 (2.08; 25.2)	0.57 ^α^	0.16; 0.81	315 (222; 400)	335 (214; 419)	19.0 (−50.4; 56.9)	0.72 ^α^	0.39; 0.88
Water only	4	0	0	-	-	-	0	0	-	-	-	102 (41.4; 227)	162 (70.6; 259)	37.3 (−33.1; 118)	1.00 *	0.95; 1.00

Significant values for r indicated with symbols: * ≤0.001 and ^α^ ≤0.05.
